# Draft genome sequence of *Nitrobacter vulgaris* DSM 10236^T^

**DOI:** 10.1128/mra.00305-24

**Published:** 2024-07-11

**Authors:** Mark Soghomonian, Angela Soghomonian, Matthew Escobar, Vera Thiel, Markus Göker, Natalia Ivanova, Rekha Seshadri, Kalyani Maitra

**Affiliations:** 1 Department of Chemistry and Biochemistry, California State University, Fresno, California, USA; 2 Department of Biological Sciences, California State University, San Marcos, California, USA; 3 Leibniz Institute DSMZ, German Collection of Microorganisms and Cell Cultures, Braunschweig, Germany; 4 DOE Joint Genome Institute, Lawrence Berkeley National Laboratory, Berkeley, California, USA; Indiana University, Bloomington, Indiana, USA

**Keywords:** *Nitrobacter vulgaris*, chemolithotrophy, draft genome

## Abstract

Here, we report the draft genome sequence of *Nitrobacter vulgaris* DSM 10236^T^, a nitrite-oxidizing bacterium isolated from a sewage system in Hamburg, Germany. The genome is 4.3 Mb in size with 4,585 predicted genes, including the full complement of genes necessary for growth on nitrite (*narK, nxrA, nxrB, nxrC,* and *nxrD*).

## ANNOUNCEMENT

Nitrite-oxidizing bacteria of the genus *Nitrobacter* play essential roles in nitrogen cycling in both terrestrial and aquatic environments. They are facultative lithoautotrophs that can grow in the presence or absence of oxygen (1 – 3). *Nitrobacter vulgaris* is a Gram-negative mesophile that has been isolated from many environments, including freshwater and soil (4). To date, genome sequencing has been performed on only one strain of *N. vulgaris* (Ab_1_) (5). The type strain *N. vulgaris* DSM 10236^T^ (also known as *N. vulgaris* strain Z^T^) was isolated from a Bauersberg waterworks sand filter in Hamburg, Germany (4). The genome sequence of *N. vulgaris* DSM 10236^T^ will support further study of its role in the nitrogen cycle.

*N. vulgaris* DSM 10236^T^ was grown in mixotrophic *Nitrobacter* medium DSMZ M.756a [https://mediadive.dsmz.de/medium/756a] at 28°C for 10 days. Genomic DNA was extracted using the MasterPure Gram-positive DNA Purification Kit (Lucigen) and sent to the Department of Energy, Joint Genome Institute for sequencing.

An Illumina short-insert DNA library was prepared with a PerkinElmer Sciclone robotic liquid handling system using a Roche KAPA Biosystems library preparation kit. DNA (200 ng) was sheared to 300 bp using a Covaris LE220, size-selected by double-SPRI, and then end-repaired, A-tailed, and ligated with Illumina-compatible sequencing adaptors containing a unique molecular index barcode. The library was quantified using KAPA Biosystems’ next-generation sequencing library qPCR kit and run on a Roche LightCycler 480 real-time PCR instrument. The library was then multiplexed with other libraries, and the pool was sequenced on an Illumina NovaSeq 6000 using NovaSeq XP v1 reagent kits (Illumina), S4 flow cell, following a 2 × 150 indexed run recipe. In total, 17,899,282 sequence reads were generated. Raw sequences were quality filtered using BBTools v.38.86 per JGI standard operating practice (SOP) protocol 1061 (6), producing 1,499,468,893 bp of sequence. The filtered and normalized reads were assembled using SPAdes (version v3.13.0) with the assembly parameters ––phred–offset 33 ––cov–cutoff auto –t 16 –m 64 ––careful –k 25,55,95 (7). Contigs with lengths <1 kb were discarded (BBTools reformat.sh: minlength). The final draft assembly was then annotated using the IMG Annotation Pipeline v.5 (8) (Table 1).

**TABLE 1:** Genome features of *Nitrobacter vulgaris* DSM 10236^T^

Total scaffold sequence length (bp)	4,293,395
Number of contigs	98
Contig N_50_ (bp)	110,847
Average fold coverage (x)	349
GC content (%)	59.5
Total genes	4,585
Protein-coding genes	4,491
rRNA genes	3
tRNA genes	61
JGI IMG/M taxon ID	2829791209
NCBI WGS accession number	JAVDPZ000000000.1
NCBI BioProject accession number	PRJNA583244
NCBI SRA accession number	SRR10872729
NCBI BioSample number	SAMN13172834

Genome analyses were performed using IMG/M (9). The genome sequence of *N. vulgaris* DSM 10236^T^ has a pairwise average nucleotide identity of 98.8% and 86.5% with the sequences of *N. vulgaris* Ab_1_ and *N. hamburgensis* X14, respectively (10). The genome contains all genes required for chemolithotrophic growth on nitrite (*narK, nxrA, nxrB, nxrC,* and *nxrD*), and its nitrite-oxidizing enzyme (NXR) operon is organized identically to the NXR operon in *N. hamburgensis* X14 and *N. vulgaris* Ab_1_ (1). Interestingly, *N. vulgaris* DSM 10236^T^ appears to be the only *Nitrobacter* genome (of seven sequenced to date) with a predicted nitrous oxide reductase gene (*nosZ*, JGI gene ID 2829793416). It is located in an operon containing a *nosR* nitrous oxide reductase transcriptional regulator and a nitrous oxidase accessory protein. These genes are typically associated with denitrifying bacteria, and therefore further research is needed to explore possible connections between *N. vulgaris* DSM 10236^T^ and denitrification (11).
